# The Analgesie Effects of Rapid and Forcible Respiration

**Published:** 1880-08

**Authors:** 


THE ANALGESIE EFFECTS OF RAPID AND FORCIBLE RESPIRATION.—Under this caption, Dr. Benjamin Lee, of Philadelphia, presents some very interesting facts in a recent issue of the *Medical Times*, of that city, going to show the correctness of Dr. Adinell Hewson's experience with this method in surgical operations, a few years ago. The matter is one of much importance, and should claim the attention of all surgeons and dentists, where brief operations are required to be performed. The procedure is exceedingly simple, and is unattended with danger. It consists of *rapid and forced respiration* for a certain length of time, during which the operation is performed, or the tooth removed. In some of the reported cases, no pain whatever was experienced from the cutting instrument, or use of the forceps. In a few cases, it seems that slight giddiness occurs after the rapid breathing has ceased, but there appears to be *absolute immunity* from danger in the procedure. How the resulting insensibility to pain is effected or brought about, through the process of rapid and forced respiration, we are unable to say, but the physiologists will doubtless give us a satisfactory solution of the mystery ére long.

